# Supplementary material for: DMRforPairs: identifying Differentially Methylated Regions between unique samples using array based methylation profiles
Source: BMC Bioinformatics. 2014 May 15;15:141. doi: 10.1186/1471-2105-15-141 (PMC4046028; doi:10.1186/1471-2105-15-141)
Supplement: Additional file 2 — DMRforPairs output for the comparison of A431-MCF7 and NA17018-NA17105. Please start from the HTML files in each folder. Available via the BMC Bioinformatics website. [file 1471-2105-15-141-S2.zip › 1394847754114233_MOESM2_ESM/A431_MCF7/figures/11131.pdf]

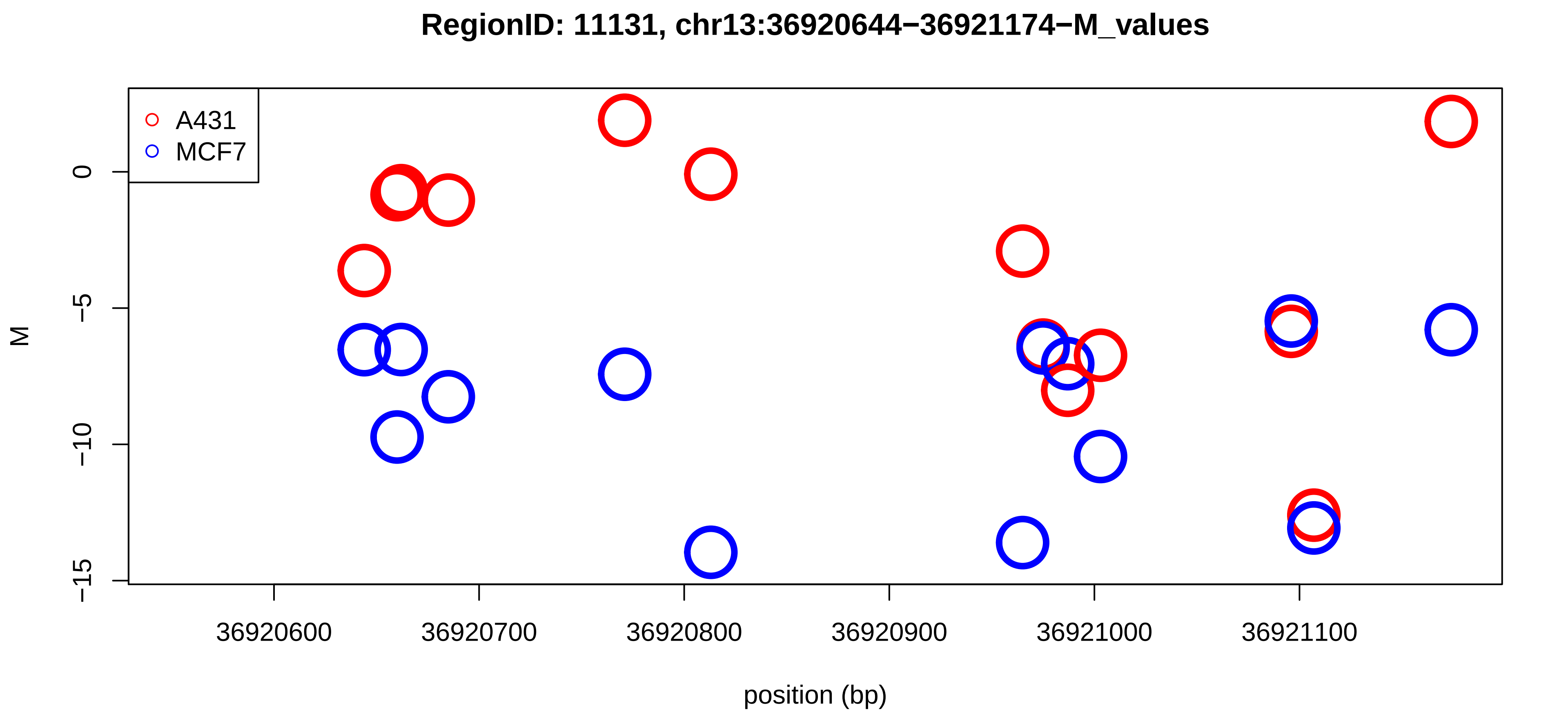
RegionID: 11131, chr13:36920644−36921174−M_values
A431
MCF7
M
0
−5
−10
−15
36920600
36920700
36920800
36920900
36921000
36921100
position (bp)

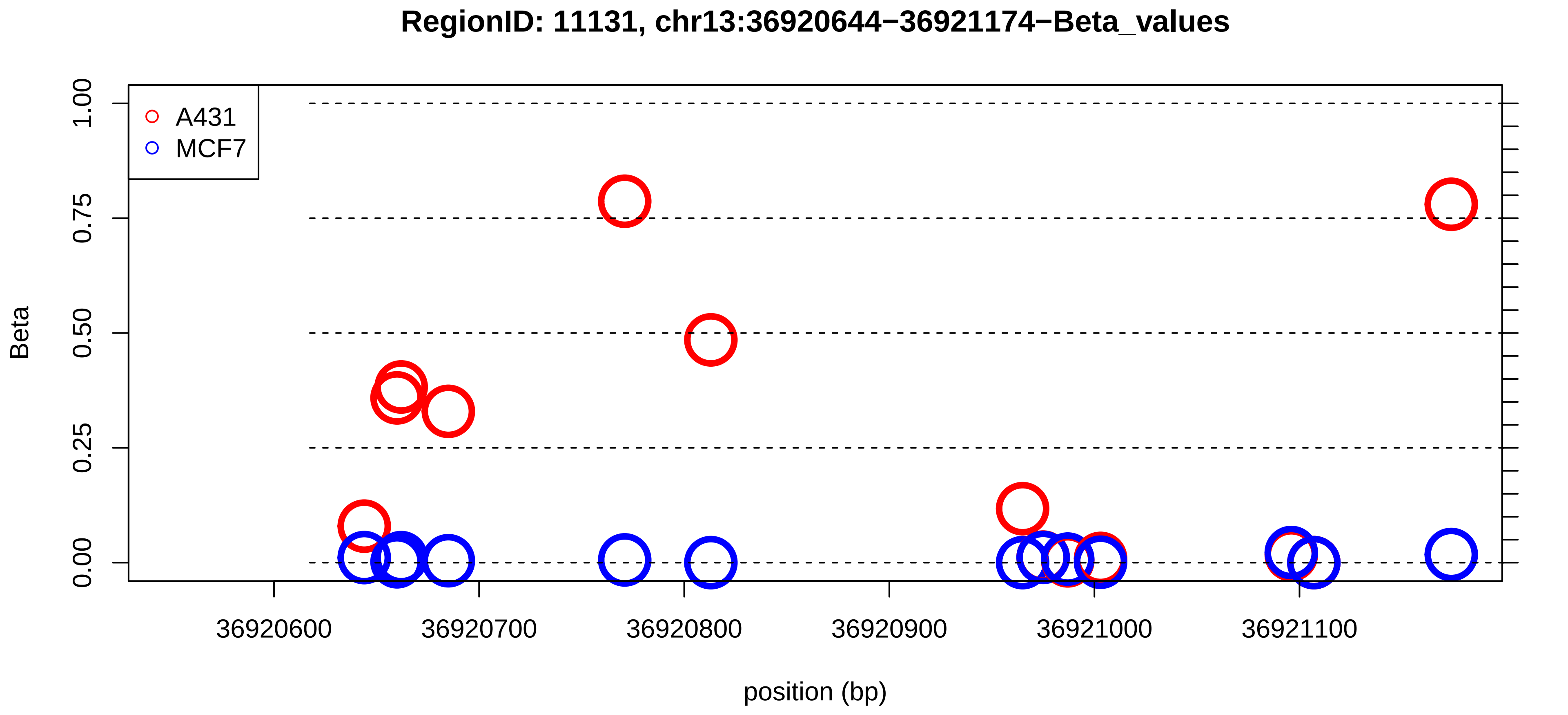
RegionID: 11131, chr13:36920644−36921174−Beta_values
A431
MCF7
Beta
1.00
0.75
0.50
0.25
0.00
36920600
36920700
36920800
36920900
36921000
36921100
position (bp)

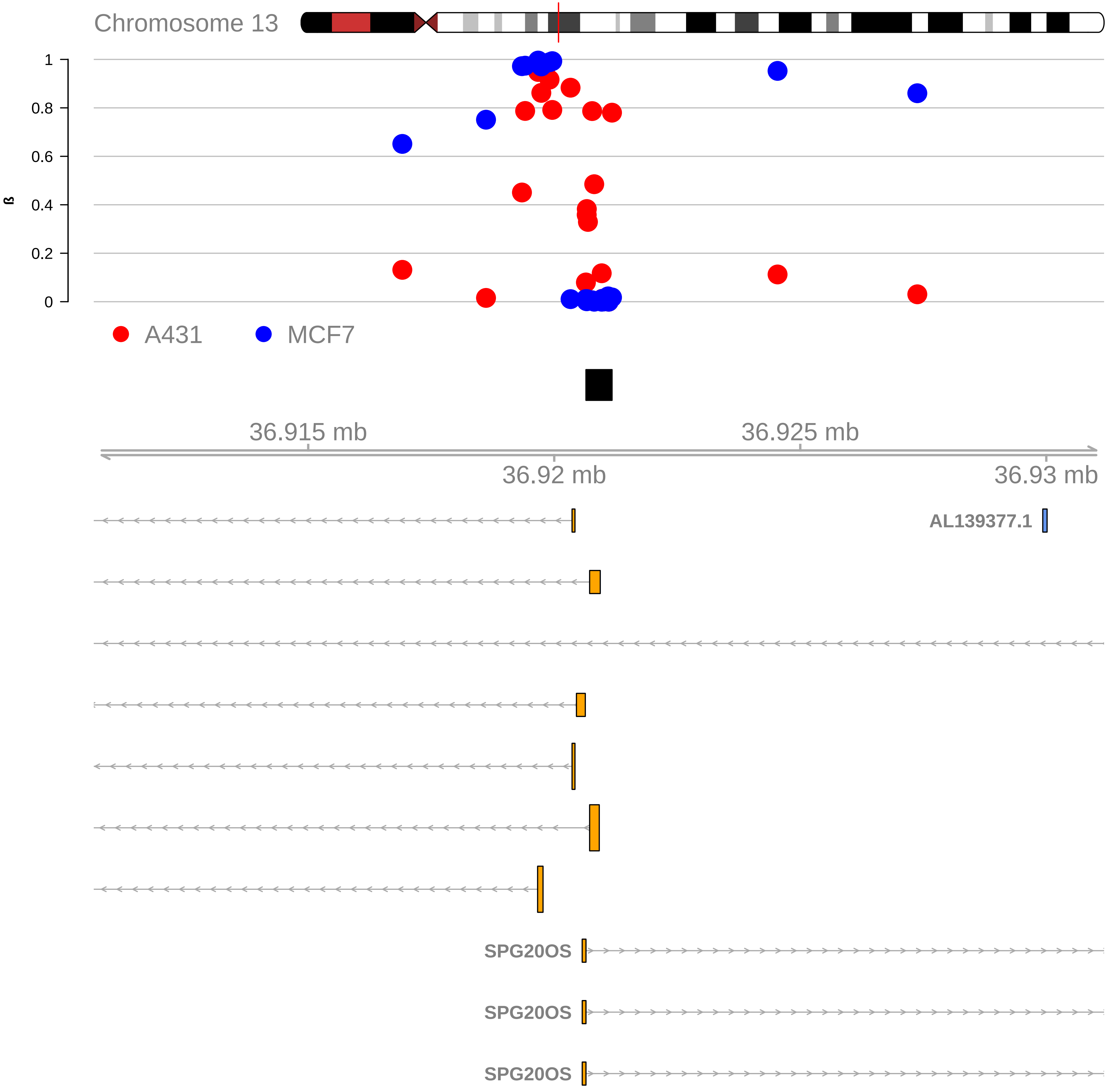

Chromosome 13
1
0.8
0.6
0.4
0.2
0
ß
A431
MCF7
36.915 mb
36.925 mb
36.92 mb
36.93 mb
AL139377.1
SPG20OS
SPG20OS
SPG20OS
